# MgrB Mutations and Altered Cell Permeability in Colistin Resistance in *Klebsiella pneumoniae*

**DOI:** 10.3390/cells11192995

**Published:** 2022-09-26

**Authors:** Polly Soo-Xi Yap, Wan-Hee Cheng, Sook-Keng Chang, Swee-Hua Erin Lim, Kok-Song Lai

**Affiliations:** 1 Jeffrey Cheah School of Medicine and Health Sciences, Monash University Malaysia, Bandar Sunway 47500, Selangor, Malaysia; 2 Faculty of Health and Life Sciences, INTI International University, Persiaran Perdana BBN, Putra Nilai, Nilai 71800, Negeri Sembilan, Malaysia; 3 Health Sciences Division, Abu Dhabi Women’s College, Higher Colleges of Technology, Abu Dhabi 41012, United Arab Emirates

**Keywords:** colistin resistance, *Klebsiella pneumoniae*, *mgrB*, PhoP/PhoQ, membrane permeability

## Abstract

There has been a resurgence in the clinical use of polymyxin antibiotics such as colistin due to the limited treatment options for infections caused by carbapenem-resistant Enterobacterales (CRE). However, this last-resort antibiotic is currently confronted with challenges which include the emergence of chromosomal and plasmid-borne colistin resistance. Colistin resistance in *Klebsiella pneumoniae* is commonly caused by the mutations in the chromosomal gene *mgrB*. MgrB spans the inner membrane and negatively regulates PhoP phosphorylation, which is essential for bacterial outer membrane lipid biosynthesis. The present review intends to draw attention to the role of *mgrB* chromosomal mutations in membrane permeability in *K. pneumoniae* that confer colistin resistance. With growing concern regarding the global emergence of colistin resistance, deciphering physical changes of the resistant membrane mediated by *mgrB* inactivation may provide new insights for the discovery of novel antimicrobials that are highly effective at membrane penetration, in addition to finding out how this can help in alleviating the resistance situation.

## 1. Introduction

In the post-antibiotic era, where the emergence of carbapenem-resistant Enterobacterales (CRE) has been reported worldwide, colistin is increasingly being prescribed as a last-resort antibiotic due limited treatment options [1]. Colistin (polymyxin E) exerts bactericidal activity against most of the Gram-negative pathogens via mechanisms involving the outer membrane (OM) disruption and the neutralisation of lipopolysaccharides. The OM of Gram-negative bacteria comprises an asymmetric and compositionally unique bilayer, with phospholipid being the inner leaflet and outer leaflet studded with lipopolysaccharides (LPS) or lipooligosaccharides (LOS) [2]. The OM plays a key role as a permeability barrier against various toxic compounds including antibiotics and detergents. Regulations of the OM barrier are triggered by environmental signals; they play a significant role in promoting antimicrobial resistance by shielding the bacteria from toxicity and selectively allowing for the uptake of the nutritional compounds required for the bacteria through porins [3]. Additionally, the bacterial efflux pump systems that traverse both the cytoplasmic and outer membranes are thought to be synergistically working with the OM in conferring antibiotic resistance [4].

While the majority of the antibiotics such as beta-lactams rely on the pore-forming porins to pass through the OM for intracellular processes, the low permeability of the bacterial OM has been identified as a challenging barrier for antibiotic sensitivity. In order to overcome these obstacles, there is a need for research to shift focus on antimicrobials with OM-targeting properties. Although the clinical use of OM-targeting colistin has been revisited, the unwelcome trends of colistin resistance among the Gram-negative bacteria followed soon after this, demanding global attention. Since the discovery of mobilised colistin resistance (*mcr*) genes, which cause lipid A modification [5], a wide variety of colistin resistance related to horizontal gene transfer has been described [6]. The effects of *mcr* expression on the global physical properties of bacterial membranes were previously discussed elsewhere [7]. Resistance to colistin can also arise through mutations in the chromosomal genes that alter the membrane properties. The modifications of the PmrA/PmrB and PhoP/PhoQ two-component systems and the inactivation of the *mgrB* gene (a regulator of the PhoP/PhoQ system) are known to be majorly involved in colistin resistance via LPS modification [8]. Among these, *mgrB* mutations seem to be common and increasingly reported in *Klebsiella pneumoniae* as compared to other Enterobacterales [9,10,11,12]. In *K. pneumoniae, mgrB* mutations which involve substitution, disruption, or inactivation have been identified as playing a prominent role in mediating colistin resistance [8].

Many studies have focused on the genetic causes of *mgrB* inactivation, but there are limited studies focused on the physical changes of the resistant membranes [12]. Thus, the present review intends to outline the current understanding of *mgrB*-associated membrane changes among the *K. pneumoniae* mutant, which may provide insights for future studies in membrane-targeting antimicrobial research.

## 2. Action of Colistin on Bacterial OM

The antibacterial activity of colistin targets the OM; as such, it is of a narrow spectrum and is potent mostly against Gram-negative bacteria, but not Gram-positive bacteria. It is active against most of the Enterobacterales including *Klebsiella* spp., *E. coli, Salmonella* spp., *Enterobacter* spp., *Citrobacter* spp., and *Shigella* spp., but *Proteus* spp. and *Serratia* spp. are intrinsically resistant to colistin. Categorised as cationic antimicrobial peptides (CAMPs), the colistin compound is positively charged and thus binds to the negatively charged phosphate groups of lipid A on the LPS via electrostatic interaction. The lipid A is an important building block of LPS which plays a critical role in maintaining bacterial permeability. Colistin acts competitively by displacing the divalent cations of calcium (Ca^2+^) and magnesium (Mg^2+^)—ions that otherwise help in stabilising the LPS molecules [13]. This leads to a series of processes of the loss of membrane integrity, the loss of the osmotic integrity of the cell membrane, the leakage of cell contents, and, subsequently, cell death [14]. Although LPS has been identified as the initial target of colistin activity, the exact mode of action of colistin remains unclear. Furthermore, colistin also exerts anti-endotoxin activity of lipid A through binding and neutralising the LPS molecules [13]. Mularski et al. (2016) examined the effect of coslitin on the OM of wild-type *K. pneumoniae* using cryo-electron microscopy (cryo-EM). Cryo-EM presented the clear visibility of the cytoplasmic membrane, peptidoglycan, OM, and fimbriae of the bacterial cell. The wide-type cells showed significant membrane damages after 2 h of exposure to colistin, including OM rupture, peptidoglycan discontinuity, and membrane blebs [15]. It has been demonstrated that the membrane damage by polymyxin molecules occurs in a concentration-dependent manner. At high polymyxin concentrations, the antibiotic molecules will form aggregates at the bacterial surface, leading to large physical defects [7]. Due to the non-specific nature of polymyxin interactions with membranes, polymyxins are avidly bound to the brush-border membrane of the kidney cells, leading to its undesired nephrotoxicity. Thus, attempts have been made to add antioxidants to the peptide molecules with the aim of suppressing polymyxin-induced membrane damage [16].

### Overview of Colistin Resistance in K. pneumoniae

Before we dive into the profound mechanism of *mgrB* gene mutations leading to colistin resistance in *K. pneumoniae*, an overview of other molecular resistance mechanisms identified will be discussed here and summarised in Table 1. Kim et al. (2014) reported that the PmrA/PmrB and PhoP/PhoQ two-component systems are upregulated in *K. pneumoniae* upon exposure to colistin. The upregulation of *pmrA/pmrB* can be caused by mutations in *pmrA* or *pmrB,* followed by the upregulation of *pmrC* and the *arnBCADTEF* operon, which subsequently results in the synthesis of L-Ara4N (4-amino-4-deoxy-_L_-arabinose) and PEtn (phosphoethanolamine) to lipid A [17]. The crosstalk between another two-component system, QseB/QseC, with PmrA/PmrB in *Escherichia coli* has been elucidated; however, the role of QseB/QseC in conferring colistin resistance in *K. pneumoniae* is not well characterised [18]. Nevertheless, recent studies showed that alterations in QseC contributed to polymyxins resistance [19,20]. Gene transformation experiments also proved that mutations in *yciM* and *lpxM* produced cells with decreased colistin susceptibility [19,21]. The role of *yciM* and *lpxM* in contributing to colistin resistance still needs to be mechanistically elucidated. It has been suggested that both mutations are associated with LPS production and lipid A modification because *yciM* is responsible for LPS biosynthesis regulation in *E. coli* [22], while *lpxM* encodes for lipid A acyltransferase [21]. Additionally, the CrrA/CrrB two-component system’s crosstalk with the PmrA/PmrB regulatory pathway has also been proposed to be mediated by the connector protein CrrC [23]. Mutations in *crrB* leading to CrrC expression have been found to result in reduced colistin susceptibility [19,23]. Apart from LPS and lipid A-associated resistance mechanisms, efflux pumps in *K. pneumoniae*, including AcrAB and KpnEF, can also be responsible for reducing colistin sensitivity. The *acrB* knockouts and *kpnEF* mutants have been demonstrated to display restored sensitivity towards a wide range of antibiotics, including polymyxin [24,25].

Before the discovery of the plasmid-mediated colistin resistance gene, *mcr-1,* which was reported in 2015 [5], chromosomal mutations were the only known mechanisms for acquired colistin resistance. The *mcr-1* gene encodes for the PEtn transferase, which adds the PEtn residue to the lipid A moiety. The gene was initially discovered from the isolates from food animal farms, and it has since been identified to be circulating among the Enterobacterales. *K. pneumoniae* was one of the initial species that carried *mcr-1* [5]. Since then, nine other *mcr* genes (*mcr-2* to *mcr-10*) have been discovered and disseminated worldwide [26]. So far, *mcr-1, mcr-3, mcr-7, mcr8*, and *mcr-10* have been reported in *K. pneumoniae* [27,28,29]. A cell morphology study showed that cells overexpressing *mcr-1* presented varying marked increases in the thickness and density of the cell envelopes as compared with cells carrying empty plasmid [30,31]. The increased expression of *mcr-1* was also concomitantly observed with a reduced cell growth rate and viability. This observation has been suggested to be related to the cell fitness and survival [31].

In an induction experiment using recombinant *E. coli*, it was observed that higher expression levels of *mcr-9* genes were induced by sub-inhibitory concentrations of colistin, and this inducible expression was shown to be related to the QseC-QseB two-component system [20]. While both of the *mgrB* and *mcr-1* genes confer colistin resistance through LPS modifications, Zhu et al. (2021) demonstrated that the coexistence of the *mcr-1* gene and chromosomal mutations posed a fitness cost of the *K. pneumoniae* mutant. The study reported that *mcr-1-*negative strains harboured mutations in *phoQ* and *mgrB* more frequently, while *crrA* and *pmrB* mutations occurred more frequently in the *mcr-1-*positive strains [32]. A cloning study demonstrated that the *mcr-1* gene has no impact on colistin resistance when it coexists with the inactivated *mgrB* gene in clinical *K. pneumoniae* [33]. The finding of this experiment suggests no synergistic effect of both genes, except for the leading role played by *mgrB* inactivation in conferring colistin resistance.

The biological cost of colistin resistance is associated with bacterial growth rates, virulence, and transmissibility, which consequentially influence the evolution of antibiotic resistance. The insertional inactivated *mgrB* mutants were found to be stable even without the presence of antibiotic selection, and did not cause any significant fitness cost to the bacterial host experimentally [9]. A further epidemiological observation using the murine gut colonisation model corroborated that the reduced biological cost of *mgrB* mutants is an important mechanism for enhanced survival outside the host and host-to-host transmission in *K. pneumoniae* [34]. The consequences of *mgrB* mutation were further underscored by an experiment using a waxworm infection model to demonstrate that inactivated MgrB contributed to the heightened hypervirulence of *K. pneumoniae* [35].

**Table 1:** Overview of colistin resistance in *K. pneumoniae*.

	Resistance Mechanism	Genes Involved	References
Chromosomal-mediated	Lipid A modification with L-Ara4N addition	*arnBCADTEF* operon	[9]
	Lipid A modification with PEtn	*pmrC*	[17]
	LPS biosysnthesis	yciM *	[19]
	Activation of LPS-modifying operation in the two-component systems	*pmrA/pmrB* *phoP/phoQ* qseB/qseC * crrA/crrB *	[9,18,23,36]
	Inactivation of negative feedback regulator of the PhoP/PhoQ system	*mgrB*	[8,9]
	Increased lipid A acylation	lpxM *	[21]
	Efflux pump	*acrAB, kpnEF*	[24,25]
Plasmid-mediated	Lipid A modification with PEtn	*mcr* genes	[5]

* denotes that the roles of the genes still need to be fully elucidated.

## 3. MgrB as a PhoP/PhoQ Regulator

Olaitan et al. (2014) observed that mutations in *mgrB* contribute more in colistin resistance among the *K. pneumoniae* compared to the mutations involved in other two-component systems, such as *pmrA/pmrB* and *phoP/phoQ* [8]. In order to ascertain the importance of these two-component systems in *mgrB-*mediated lipid A modifications, the lipid A moieties synthesised by *mgrB-phoQ* and *mgrB-phoQ-pmrAB* mutants as well as the mutants complemented with *phoPQ* were compared. It was found that the previous mutants (*mgrB-phoQ* and *mgrB-phoQ-pmrAB*) resembled that of the wild-type, while the mutants complemented with *phoPQ* resembled the lipid A produced by the *mgrB* mutant. This further confirmed that the inactivation of the *mgrB* gene giving rise to colistin resistance can be PhoPQ- but not PmrAB-dependent [35]. MgrB is a small, 47-amino acid regulatory transmembrane protein which negatively regulates the PhoP/PhoQ system in Gram-negative bacteria [36]. A pioneering study by Poirel et al. (2015) demonstrated that a premature stop codon in the sequence of *mgrB*, leading to truncated MgrB with only 29 amino acids, is a key target for colistin resistance in *K. pneumoniae* [11]. However, it is understudied how such a small protein exerts a great impact on the PhoP/PhoQ pathway. Previous work has suggested that MgrB acts by directly inhibiting PhoQ histidine kinase activity, thereby modulating a negative feedback loop [36,37]. Studies have suggested that MgrB may interact with other proteins aside from PhoQ for stress responses. In acidic conditions, the inactivation of MgrB resulted in the increased accumulation of RpoS, the stress regulator, through the regulation of IraM expression [34,38]. In the absence of functional MgrB, PhoQ over-activation and the accumulation of RposS were found to be significant contributors during the environmental survival of *K. pneumoniae* [34].

In *E. coli*, a recent work suggested that the MgrB transmembrane and periplasmic regions are necessary membrane anchors establishing the physical interaction with PhoQ but are not sufficient to inhibit PhoQ. Further investigation also pinpointed a number of functionally important residues spread across the protein that are important for PhoQ’s inhibitory function. The study also tested the expression levels of MgrB orthologs from *K. pneumoniae* and other related enterobacterial species in *E. coli.* As expected, MgrB from *K. pneumoniae, Serratia* spp., and *Salmonella Typhimurium* showed comparable reporter activities to that of *E. coli* MgrB, indicating that the MgrB orthologs are generally highly conserved [39]. Interestingly, although MgrB orthologs are found several Enterobacterales, further studies are clearly needed to decipher how mutations in *mgrB* have emerged as a predominant mechanism for acquired colistin resistance, as compared to other species, especially *E. coli.*

### 3.1. MgrB-Dependent Lipid A Modifications

**Figure 1:**
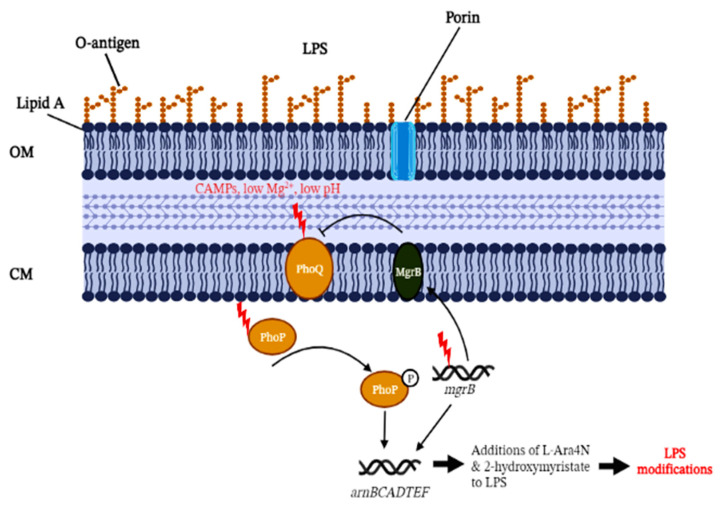
Summarised colistin resistance mechanism associated with MgrB inactivation in *Klebsiella pneumoniae.* PhoQ is stimulated by low extracellular cationic magnesium or cationic antimicrobial peptides (CAMPs) under low pH conditions, leading to increased PhoP phosphorylation. This, in turn, drives the transcription of *mgrB.* The accumulation of MgrB results in a negative feedback loop to inhibit the kinase activity of PhoQ, which subsequently suppresses PhoP phosphorylation. Mutations (denoted by red-coloured thunder symbols) in *mgrB* or MgrB inactivation disrupting the PhoP/PhoQ pathway eliminates this partial adaptation. The disruption of *mgrB* can mediate the activation of the *arnBCADTEF* operon for the addition of L-Ara4N to lipid A. Phosphorylated PhoP can also directly activate the *arnBCADTEF* operon without other PmrA/PmrB-activated proteins in *K. pneumoniae.* OM, outer membrane; CM, cytoplasmic membrane, LPS, lipopolysaccharide, L-Ara4N, 4-amino-4-deoxy-_L_-arabinose. (Created with BioRender.com, accessed on 16 August 2022).

As depicted in Figure 1, the disruption of *mgrB* can up-regulate the PhoP/PhoQ system and the operon *arnBCADTEF*, which are responsible for the addition of the L-Ara4N moiety to lipid A [9]. However, this notion was not mechanistically proven until Kidd et al. (2017) combined biochemistry and genetic testing to demonstrate PhoPQ-governed lipid A remodelling induced by the inactivation of the *mgrB* regulatory gene. The work also highlighted key modifications to the lipid A moiety with the additions of L-Ara4N and 2-hydroxymyristate [35]. The characterisation of L-Ara4N is relatively well studied, but the role of 2-hydroxymyristate needs to be further elucidated. The addition of L-Ara4N to lipid A structures was also confirmed using mass spectrometry, in congruence with the observed genetic changes of *mgrB* genes. However, it was observed that different *mgrB* gene variants or types of loss-of-function (e.g., insertional inactivation, deletion, or premature stop codon) did not contribute significantly to the variability of lipid A on the mass spectrometry profiles [40]. A similar observation was also reported in Kidd et al. (2017) with clinical colistin-resistant *mgrB* mutant strains isolated from different individuals, geographical locations, resistance mechanisms, and *mgrB* gene mutations; all mutants showed similar lipid A species under the mass spectrometry [35].

The bacterial surface charge is a physiochemical barrier that regulates the interaction of bacteria with ions, particles, and surfaces, thus affecting the bacterial cell’s susceptibility to antibiotics. The direct measurement of the surface charge density is experimentally laborious. Thus, bacterial zeta potential measurement has been adopted to infer the surface charge through the electrophoretic mobility of cells in the local aqueous environment [41]. The addition of L-Ara4N to the negatively charged phosphate groups of lipid A was proposed to reduce the net-negative charge of the OM of *K. pneumoniae.* The resultant net positive charge in the modified LPS reduces its affinity to the cationic colistin [42]. A similar hypothesis on the association between *mcr-*expression and surface charge alternations has been well characterised on *mcr-1-*bearing *E. coli* and *Aeromonas veronii* strains through zeta potential measurement and LPS analysis [43]. However, Al-Farsi et al. (2019) did not observe an altered surface charge on colistin-resistant *K. pneumoaniae* due to *mgrB* insertion through zeta potential measurement. This suggests that *mgrB* insertions do not alter the bacterial surface charge in colistin-resistant *K. pneumoniae*, but they could be involved in hydrophobic interactions or the loss of O-antigens in LPS. This hypothesis is raised based on the observation that all 17 clinical *K. pneumoniae* isolates used in the study shared identical virulence genes and bacterial capsules but harboured different O-serotypes [44]. However, this hypothesis has not been mechanistically proven.

### 3.2. MgrB-Dependent Altered Cell Morphology

Studies that captured the morphological difference of the *mgrB* mutants are scarce. Formosa et al. (2015) demonstrated that *K. pneumoniae mgrB* mutant cells were presented with a thicker extracellular capsule tightly bound to the bacterial cell wall, whereas colistin treatment removed the capsule from the susceptible strain under the atomic force microscopy (AFM). When the susceptible strain was treated with colistin, the extend force curves recorded did not show a spike, suggesting that the colistin treatment caused the rupture of the bacterial capsule. On the other hand, the extend force curves displayed an apparent loss of the capsule organisation of the *mgrB* mutant cells upon colistin treatment, too. However, according to further investigation on the nanomechanical properties of the cells, the stiffness values of the cell wall increased in the colistin-resistant strains when treated with colistin as compared to the cells in native conditions. The AFM images showed that the *mgrB* mutant cells were wider than the susceptible cells, indicating that the latter had a capsule with multiple layers and different morphologies. Such observations led to a previously undescribed capsule phenotype which could probably be linked to *mgrB* inactivation. The study reported no variation in the expression of genes associated with the LPS synthesis pathway [45]. The experiment, however, confirmed that *mgrB* mutation exerted direct consequences on the structural organisation of the colistin-resistant strain.

In order to assess the difference in capsular polysaccharide (CPS) production between the wild-type and *mgrB* mutants, Bray and colleagues measured the amount of uronic acid, which is the major component of CPS in *K. pneumoniae.* Under in vivo experimental conditions, the team observed that *mgrB* mutants had reduced CPS production, which is associated with enhanced mucin binding ability, facilitating their eventual gastrointestinal tract clearance. Further testing on the cell survivability outside the host through solid surface starvation survival experiments showed that *mgrB* mutants with reduced CPS had significantly higher survival rates than those of the wild-type strains. It has been deduced that the LPS fatty acid composition modification observed in the mutant cells may have contributed to the altered membrane rigidity and subsequently benefited the environmental survival. Taken together, the study demonstrated that the MgrB-mediated dysregulation of the PhoPQ system had a fitness cost in gut colonisation but enhanced cell adaptation and survivability outside the host, which benefit the bacterial host-to-host transmission [34].

Under transmission electron microscopy (TEM), the *K. pneumoniae mgrB* mutant with IS1 transposase insertion showed a thickened cell envelope compared to the colistin-susceptible cell [46]. In another transcriptomic morphological study, *K. pneumoniae* with *phoQ* and *crrB* mutations were observed to retain the membrane integrity in addition to fimbriae production upon colistin exposure. The TEM observations were corroborated with the transcriptomic data, in which the overexpression of OM proteins, *slyB* and *yfgL,* was identified to be responsible for increased membrane permeability. The detection of fimbriae was also visible under the TEM, and this was correlated with the upregulation of *fimACDFG* [47]. Type 1 fimbriae are crucial virulence factors found in the majority of enterobacterial species, which had been suggested to also play a role in antibiotic evasion aside from adhesion [48]. In the same study, other notable increased genes, including *mgtA, fepAC*, and the genes of the FeCT operon, which are associated with ion transportation, had been suggested to be related to dark ion-dense granules accumulation under TEM observations. The work also led to the observation of extracellular polysaccharide (EPS) in the TEM images, but there were no genetic or transcriptomic data identified to explain this phenotype [47]. This experiment also suggested that, although *mgrB* may play an important role in the PhoP/PhoQ regulatory system, altered cell membrane morphology could be attributed to mutations in *phoQ* or *crrB* alone.

For bacterial cell morphology experiments, it is important to be cautious regarding the modification of the cell ultrastructure due to the inevitable steps of dehydration during sample processing. Bacterial cells under SEM or TEM often show significant cytoplasm shrinkage, which may not truly reflect cells’ ultrastructure in vivo [5]. In comparison, cryo-EM eliminates the dehydration stress that could result in unintended bacterial cell ultrastructure modifications [15]. The electron microscopy techniques typically provide magnification of the cell in two dimensions (x and y), while the AFM provides magnification in three dimensions (x, y, and z). Thus, it is a very useful tool for the mechanical measurements of altered membrane topology at a nanometric level. Ierardi and colleagues demonstrated an innovative approach to utilise AFM images in distinguishing between colistin-sensitive and colistin-resistant *K. pneumoniae* strains [49]. Owning to the relatively lower cost of AFM as compared to SEM and TEM, the procedure opens potential possibilities for the rapid detection of antibiotic resistance in bacteria based on cell morphology by AFM.

Given that the antibiotic resistance mechanisms in *K. pneumoniae* are often related to the OM permeability, evidence on the direct observation of morphological alterations with microscopic techniques is disproportionally lacking in comparison to other phenotypic and genotypic studies. The results from SEM, TEM, or AFM imaging enable researchers to combine the genetic data with direct consequences on the organisation of bacterial cell ultrastructure, including OM, capsule, fimbriae, etc., in response to antimicrobial agents’ exposure. Furthermore, there have been limited studies associating the types of genetic mutation or insertion sequences of the *mgrB* gene with the altered physiochemical properties observed in the LPS and lipid A. These data would contribute to the further characterisation and enhanced understanding of the ever-changing bacterial membrane in an era of an increasing prevalence of multidrug-resistant strains.

## 4. New OM-Targeting Antibiotics

### 4.1. Polymyxin Derivatives

Combining the knowledge of colistin activity and resistance, research has focused on the similarities between colistin and other membrane-disruption antimicrobials. As a direct approach, antibiotic synergy was explored. Colistin in combination with clarithromycin was shown to have improved efficacy and survival against *mcr-*expressing isolates in vivo [50]. A possible explanation for this phenomenon is that the destabilisation of the membrane structure was initiated, leading to increased permeability to antibiotics for intracellular targets. Due to the post-antibiotic era urgency, more than USD 40 million of funding has been allocated to stimulate research into the design/discovery of polymyxin derivatives over the past few years [16]. The challenge for developing new polymyxins is not only targeting the colistin-resistant Gram-negative bacteria but also reducing the inherent toxicity of polymyxins. The N-terminal fatty-acyl moiety of polymyxin contributes greatly to the bactericidal activity; however, at the same time, it also contributes substantially to the mammalian cells [51]. Macolacin, a derivative from the polymyxin family, differs from colistin by three amino acids, and it was found to be potent against a number of colistin-resistant Gram-negative pathogens in the latest study. Despite its unclear mode of action, it was reasoned that the compound may have a different molecular target which retains its activity under modified lipid A moieties [52]. Certain polymyxin derivatives that lack direct antibacterial activity have been demonstrated to bind to LPS and damage the OM, facilitating the entry of other antibiotics into the cell [53]. Notably, SPR741 (formerly known as NAB741), a cationic derivative compound of polymyxin B, does not exert bactericidal activity itself but sensitises the bacterial cells to beta-lactam antibiotics against major Gram-negative pathogens. The peptide compound passed Clinical Phase 1 in 2017, with reduced non-clinical nephrotoxicity [53]. It is a successful model for an OM permeabiliser that sensitises colistin-resistant Gram-negative bacteria to other antibiotics.

As increasing number of research teams are focusing on developing novel polymyxin derivatives; one growing practical trend is worth noticing. Agents that cause membrane damage may be effective and valuable membrane permeabilisers to the action of other antibiotics. These include antimicrobial peptides (AMPs) and cell-penetrating peptides (CPPs) that work similarly to colistin and other CAMPs, whereby the positively charged compound will bind to the bacterial OM which is negatively charged [54]. However, cross-resistance between colistin and other CAMPs could exist, since the two share similar membrane-binding mechanisms [44]. In this case, AMPs incorporated with hydrophobic residues may help to enhance the membrane permeabilising ability since the bacterial cell surface hydrophobicity plays a crucial role in cell physiology [55]. With this notion, Velkov and colleagues had derived a series of polymyxin-like lipopeptides with hydrophobic motifs at the positions R6 and R7, specifically targeting the polymyxin resistance. The structure–activity relationship model for the lipopeptides suggested that hydrophobic modifications at the polymyxin scaffold facilitated the drug’s interaction with modified lipid A moieties. In terms of nephrotoxicity, the lipopeptide FADDI-003 did not present any histopathological damage to the kidney in a treated mice model as compared to a model treated with polymyxin B [56].

### 4.2. Hydrophobic Membrane-Active Agents

Unlike polymyxins with binding sites at lipid A or lipid II, hydrophobic antibiotics bind at different target sites for the disruption of membrane integrity. Such an approach could attenuate the consequences of lipid A modifications caused by the *mgrB* mutation. Emerging studies have also highlighted the hydrophobicity of natural products such as essential oils (EOs) as potential membrane permeabilisers. It has been suggested that the hydrophobic tail of the EOs will bind preferentially to the cell membrane’s phospholipids, thereby disrupting the membrane stability and permeability, leading to cell contents leakage [57]. However, this unspecific mechanism may render undesired cytotoxicity effects on any biological membranes, including mammalian cells [58].

Due to the inherent toxicity of polymyxins and other membrane-targeting agents, researchers are hoping to design small peptides or analogs that bind to bacterial LPS only. Compounds with different types of hydrophobic moieties were synthesised to assess the extent of their membrane perturbation, their potentiation activity, and their toxicity. For instance, Plantaricin A (PlnA) analogs were designed for this reason to bind with the LPS of Gram-negative bacteria through electrostatic and hydrophobic interactions. The binding of PlnA and LPS was suggested to destroy bridges between the LPS moieties and subsequently disrupt the OM integrity [59]. In combination treatment, the disruption of the physical integrity of the bacterial membrane by the hydrophobic agents could synergise conventional antibiotics against colistin-resistant superbugs. It was found that analogs with weak membrane permeability activity were sufficient to exert antibiotic potentiation effects [60].

### 4.3. Challenges and Future Directions

It is important for scientists and clinicians to understand the contribution of Ara4N and 2-hydroxymyristate addition to modified lipid A species in the *mgrB* mutant for careful consideration of colistin regimens for treating *K. pneumoniae* infections. The MgrB inactivation conferring a fitness cost of *K. pneumoniae* towards environmental survival could pose a silent threat for its transmission in hospital settings. Evidence of changes in the physical properties of *mgrB* mutants also suggests their resistance to disinfectants used in hospitals. Therefore, the current review also highlights the urgent consideration to include the identification of *mgrB* clones in *K. pneumoniae* in clinical microbiology laboratories.

Synergistic regimens with membrane-potentiating agents may seem to be the way forward; however, high membrane activity for antibiotic potentiation is a double-edged sword which often leads to associated cytotoxicity. The mechanism of membrane perturbation is often non-specific and remains highly elusive. The extent of extensive membrane perturbation resulting in toxicity or other potential side effects has not been stressed. Future research to identify specific and consistent binding sites of the membrane-potentiating compounds could aid solutions to advance the development of drug discovery.

## 5. Conclusions

Taken together, MgrB-dependent colistin resistance was found to affect the global physical properties of bacterial membranes in *K. pneumoniae.* The principles behind the colistin resistance conferred by *mgrB* involved increased membrane integrity and stability as well as lipid A modifications, thus resulting in a reduced affinity for the antibiotic to the cell. The current review also highlighted the relatively less described altered bacterial capsular phenotypes, which have been associated with MgrB inactivation. Given the rapid evolution of bacterial membranes, it is important for researchers to be updated on these processes so that we are better informed to design approaches aimed at combating antimicrobial resistance.
